# Financial hardship after cancer: revision of a conceptual model and development of patient-reported outcome measures

**DOI:** 10.2144/fsoa-2023-0229

**Published:** 2024-05-14

**Authors:** Salene MW Jones, Jean Yi, Nora B Henrikson, Laura Panattoni, Veena Shankaran

**Affiliations:** 1 Fred Hutchinson Cancer Center, 1100 Fairview Ave N, Seattle, WA 98109, USA; 2 Kaiser Permanente Washington Health Research Institute, 1730 Minor Ave, Seattle, WA 98101, USA; 3 University of Washington, 1959 NE Pacific St, Seattle, WA 98195, USA

**Keywords:** administrative burden, administrative hurdles, economic burden, economic wellbeing, financial burden, financial toxicity

## Abstract

**Aim:** This qualitative study refined a conceptual model of financial hardship and developed measures corresponding to model constructs. **Methods:** Eighteen women with breast cancer recruited through a comprehensive cancer center completed interviews. A qualitative framework analysis was conducted of the interviews. **Results:** Participants experienced varying levels of financial hardship. Protective factors included good health insurance, work accommodations and social support. Participants worried about cancer care costs and employment. Programs for alleviating financial hardship had high administrative burdens. Four preliminary financial hardship measures were developed: coping, impacts, depression and worry. **Conclusion:** Reducing administrative barriers to benefits could reduce financial hardship after cancer. More research is needed on the effects of out-of-network/formulary care and denials of coverage and to validate the measures.

## Plain language summary

Financial hardship is common after cancer diagnosis. This study interviewed women with breast cancer about financial hardship. Financial hardship included how participants coped with healthcare costs and reduced income. Worry and depression were also aspects of financial hardship. Administrative burdens led to financial hardship. Administrative burdens were actions patients had to take to access financial support. This study also created surveys to measure financial hardship in cancer.

## Tweetable abstract

This study revised a conceptual model of financial burden after cancer. Measures were developed for each financial burden dimension from the model. Reducing administrative hurdles for work accommodations and insurance could prevent burden.

## Abstract (plain language summary)

Financial hardship had multiple dimensions including coping behaviors, consequences, worry and depressive symptoms.

Item banks to assess each dimension of financial hardship were developed.

Good health and disability insurance, employment supports and social support were protective factors.

Administrative hurdles to accessing insurance and supports led to financial hardship.

More research is needed on the role of specific administrative hurdles in financial hardship.

## Graphical abstract

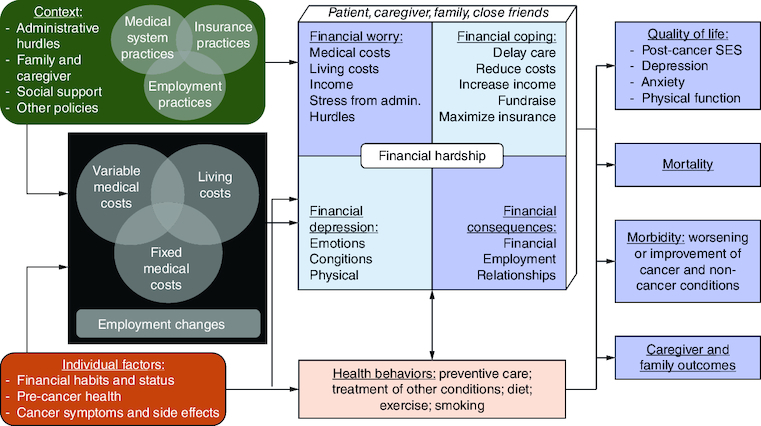

Financial hardship after cancer diagnosis is an unfortunately common occurrence. While rates of financial hardship vary by definition and measures, between 35 and 79% of people with breast cancer can experience financial hardship after diagnosis [1]. Financial hardship is also sometimes called financial toxicity when directly attributable to the cancer [2,3]. Studies have linked financial hardship after cancer diagnosis with worse quality of life [^4-7^] and earlier mortality [^8-10^]. Financial hardship after cancer is a significant public health and medical problem that requires multiple levels of intervention.

Conceptual models help researchers understand a problem and focus research for preventive strategies and help clinicians identify where they can treat a problem. Previous models of financial hardship after cancer laid a solid but mostly descriptive foundation [11,12]. There is still a need for models to fully account for the complexity of financial hardship, such as distinguishing causes of financial hardship from the experience of the patient and their family. Another need is to fully account for the experience of psychological facets of financial hardship. Our previous conceptual model [13] outlined four unique dimensions of financial hardship. First, financial coping was actions patients take to afford care and living costs [14]. Financial consequences were impacts and events that occur when a patient and their family cannot afford care or basic needs, such as bills going to collections. The psychological aspects of financial hardship were divided into financial depression, or depressive symptoms due to economic burden, and financial worry or financial anxiety about paying for medical care and living costs. Our conceptual model also distinguished causes of financial hardship (medical costs, living costs, decreases in income) from financial hardship itself.

Although several measures of financial hardship exist, challenges remain. Some measures are not cancer specific and have not been validated in cancer populations [15,16]. One measure has been developed specifically for use in cancer [17,18], but is very general with no ability to distinguish different dimensions of financial hardship such as financial coping or the psychological dimensions of financial hardship and misses these important patient burdens. Other measures such as the Socioeconomic Wellbeing scale have been developed to measure financial hardship as it aligns with quality of life [19]. Lumping all financial hardship dimensions together creates a ‘black box’ situation where mechanisms of financial hardship cannot be identified and elucidating factors causing financial hardship are more challenging. The purpose of the current study was to use patient experiences to revise our conceptual model and to begin developing measures of the four different dimensions of financial hardship.

We conducted an interview study of women with breast cancer. The interviews served two purposes. First, information on participants' experience of financial hardship was used to refine our conceptual model of financial hardship after cancer. While previous research has identified insurance coverage and employment changes as potential causes of financial hardship, we aimed to identify more specific barriers of insurance use and employment that lead to financial hardship and could be targeted by interventions. Second, interviews helped in developing item banks (collections of survey or questionnaire items) to assess each dimension of financial hardship and ensure survey items were understandable. Our revised conceptual model and proposed item banks will enable a future study that will generate psychometrically validated item banks for use in both research and clinical screening.

## Materials & methods

### Item bank development

Item banks assessing four dimensions of financial hardship were developed based on previous measures [20,21] and scientific literature reporting manifestations of financial hardship after cancer [11,12,22,23]. The investigative team reviewed the questions and revised the items for clarity and breadth. External experts provided feedback on the items. Items were mapped to the four dimensions of financial hardship from the initial conceptual model [13]. Items for financial coping and financial consequences/impacts had dichotomous yes/no response options and referenced the past year (12 months). Items for financial worry and financial anxiety had response options like the previous measure (not at all fearful, a little fearful, somewhat fearful, very fearful). Items for the financial depression and rumination bank were patterned after symptoms for major depressive disorder [24], but specifically referencing symptoms due to finances. The response options for the financial depression and rumination bank were not at all, several days, more than half the days and nearly every day.

### Participants & procedures

Participants were recruited through the Survivorship Program at an NCI-designated Comprehensive Cancer Center. Women with breast cancer who had consented to be contacted about future studies (n = 162) were emailed one to three invitations about the study. Eligibility criteria were diagnosis of breast cancer; being at least 3 months post-diagnosis; age 18 or older; able to read and speak English; able to provide informed consent; and located in the USA. Interested women responded and were sent the consent form. Interviews were then scheduled and conducted over a secure, HIPAA-compliant virtual platform. The platform allowed for video interviews or phone interviews. Each participant provided verbal informed consent at the beginning of each interview and was offered a $20 gift card for completing the interviews. The informed consent process included that de-identified direct quotes may be used in publications. Interviews were recorded and the transcription method from the virtual platform was used to create a preliminary transcript. A staff member reviewed and verified each transcript against the interview recording.

Interviews were conducted in two sets: content and cognitive. The first 13 interviews were content interviews and focused on exploring participants' experience of financial burden. For participants without significant financial burden, interviews elucidated factors that prevented financial hardship. Results from the 13 content interviews were used to revise the item banks and theoretical model. The last five interviews were cognitive interviews. For these five interviews, participants were sent the item bank questions before the interview and the interviews focused on revising the questions as well as the participants experience of financial burden. Interviews ranged in length from 45 to 60 min.

### Statement on ethics approval

The institutional review board of Fred Hutchinson Cancer Center reviewed all study procedures and determined the study was exempt from review under Category 2, Tests, Surveys, Interviews and Public Observation, under 45 Code of Federal Regulations 46, as the study only involved interviews.

### Qualitative analysis

The analysis of interview data used a deductive, framework approach [^25-28^]. The principal investigator (PI) developed an initial codebook based on the original conceptual model with input from another member of the investigative team who is a financial hardship and qualitative research expert. The PI and a second coder reviewed the codebook and then both coded three transcripts. Disparities in codes were discussed and reconciled. When the PI and the second coder could not come to consensus, the financial hardship/qualitative research expert provided resolution. The codebook was also revised to better capture participants' experience, including adding new codes and revising definitions of codes. The second coder then coded the remaining 15 transcripts, discussing any questions or quotes that were unclear with the PI. The second coder also marked important quotes that did not clearly fit into a category with an ‘other’ code. The PI reviewed all quotes in the ‘other’ code and the two coders discussed which code should be used for each quote. If the two coders were unable to reach consensus for a quote under the ‘other’ code, the financial hardship/qualitative research expert provided guidance. The PI then conducted content analysis on the coded transcript data. Atlas.ti software was used for qualitative coding and analysis.

Results from the interviews were then used to revise the item banks and conceptual model. For the item banks, sometimes participants report of financial coping strategies or financial worries was directly translated into a new item (extension on a bill). Other items were created through discussion by the investigative team of the results (bartering). The PI made a draft revision of the conceptual model based on themes from the interviews. The draft revised model was then reviewed, discussed and further revised by the investigative team. Revisions were made to improve clarity of the model and accurately reflect themes from the interviews.

## Results

Eighteen women completed interviews, with 13 content interviews followed by five cognitive interviews in 2022. Although in-person interviews were available, all participants elected to complete the interview via phone or video call. No participant had known metastatic disease (stage IV). Eighty-three percent were non-Hispanic white. The mean age of participants was 57 years at the time of the interview. Twelve (67%) were married or in a long-term relationship at the time of the interview. All participants had completed high school and at least some college. Thirteen had completed a graduate degree (master's degree or doctorate). Time since diagnosis ranged from 3 to 24 years. All participants had surgery for their breast cancer, 13 (72%) had chemotherapy and 12 (67%) had radiation treatment.

Overall, results of the interviews suggested a gradient of financial hardship and multiple factors contributing to where participants fell on that gradient (see Table 1 for example quotes). The financial hardship gradient ranged from covering minimal additional costs and tracking health insurance payments to using up savings and, at the most severe end, not paying for basic living costs and foregoing treatment due to cost. Even participants that managed to financially cope and survive financially were distressed by the amount of stress and effort required to do so. Participants with financial resources and financial literacy still had difficulty coping with the financial burden of breast cancer. Financial effects of cancer could also last decades after diagnosis. More detailed discussion of the themes is reported below.

**Table 1.** Model constructs and exemplar quotes.

Model construct	Definition	Quotes
**Causes of financial hardship**		
Employment factors	Employment practices primarily center on support for maintaining employment and income during the cancer, such as whether leave is paid or unpaid, availability of reasonable accommodations and the ease with which paid leave and accommodations can be accessed	• *“He [boss] was like just go home, you know, just those little things. Like, just go home now, no take the day. You don't have to take off hours today and if you feel good, you know, just check your email or write up a little summary of something you know and that'll count and like just those little graces.”* • *“They [workplace] were very supportive, but my health insurance was through my employment, which meant I had to stay working the whole time.”*
Insurance factors	Insurance practices includes health insurance and other types of insurance (disability, life) and encompasses requirements to access coverage and the level of coverage	• *“I was probably on the phone with them [health insurance company] once every three or four weeks trying to figure out what it was that they were doing on some of their explanation of benefits resubmitting things. Just it was really painful.”* • *“Because although I could have COBRA'd, I couldn't have afforded all my expenses.”* • *“But when I was going through this I had to go on disability. My disability insurance tried to screw me. Oh, they tried to mess with how much they were giving me…Yes, they tried to short me. Yes, it was thousands of dollars”*
Out of pocket care costs	Costs for healthcare and from treatment side effects. Medical system practices that affect costs includes how procedures are coded, billing practices such as whether a provider will send a bill to collections, locations of clinics and how many appointments a patient need	• *“Other types of therapy. You know, acupuncture, that type of thing, and that was all out of pocket.”* • *“The plastic surgery center wanted all of its money up front for the reconstruction surgery.”* • *“Clothes wise, certainly when I was in chemo, I had to get clothes that were a little bit smaller.”*
Living costs	Costs for basic needs and non-essentials (gym memberships, hobbies)	• *“And there were things that we didn't have available to us any longer that. Maybe our idea of our life together just it wasn't possible, more like travel and bigger purchases.”*
**Dimensions of financial hardship**		
Financial coping	Participants' actions to afford care and living costs	*• “We have not currently delayed retirement, but probably will be doing that.”*
Financial consequences	Financial events that happen to participants due to trouble coping	*• “I was not able to do much savings then for retirement personally.”*
Financial depression	Symptoms of depression due to finances	*• “I'm angry or mad because of money.”*
Financial worry	Worry and anxiety due to finances	*• “There was a lot of uncertainty about what things would cost.”* *• “So, the idea was would I have enough? Vacation or leave or how was I gonna do this without losing my job?”*
**Context and individual factors**		
Positive support	Resources others provide that can buffer the effects of context	*• “There was a physical fitness program that they (cancer center) had for those in chemo.”* *• “My boss even paid for house cleaning and stuff like that and they came to visit and brought food.”*
Social and caregiving context	The patient's role as a caregiver to others and the ability of caregivers to support the patient	*• “The first time I got breast cancer, I was a single mom with a 9 year old.”* *• “I was married at the time, so we had two incomes, no kids.”*
Time and location	Location of care centers and changes in causes of financial hardship over time	*• “And the bus…when I was doing chemo, I was a little bit paranoid about taking a bus with all those other potentially sick people.”*
Cancer symptom burden and financial hardship	Cancer and cancer treatment symptoms that led to financial hardship	*• “I think clearly because it was a huge swath of, you know 8-9 months of my life that I wasn't progressing at my work. Uh, it certainly has put me behind on my career track.”*
Luck	Random chance or another providing support that reduced financial hardship	*• “I was lucky in that I had a 4/10 schedule.”* *• “And I was very lucky in that the insurance I was covered by my spouse at the time was very generous and so took care of most of [the costs].”*
Individual factors	Includes financial status before the cancer; pre-cancer financial habits; pre-cancer health; cancer symptoms and treatment side effects	*• “[before the cancer diagnosis] We took vacations and saved and paid that off. So I mean, there's a hardship in the sense of- I mean, we made those personal lifestyle choices to go on vacation or do those kind of things [after cancer diagnosis].”*
**Effects of financial hardship**		
Non-financial consequences	Indirect effects of financial hardship on participants' mental, emotional and physical health	*• “Meaningful financial support would have meant that I could have the rest time I needed, the recovery time I needed.”*
Caregiver effects	Financial hardship experienced by caregivers	*• “I'm pretty sure whatever we sold off for assets was mine alone. And so in the vein of, we would have both jointly had that, had it existed in retirement, and we'd still been together, yes.”*
Health behaviors	Interaction of financial hardship and actions patients take to improve their health	*• “Definitely tried to improve my diet after diagnosis, so there is a cost I would say I tried to eat more organic, definitely more local foods.”*

### Causes of financial hardship

To inform model revisions, we examined themes around employment and costs. Costs, unless otherwise specified, is used as a term inclusive of out-of-pocket care costs, basic needs and other living costs. These factors were key causes of financial hardship in the previous model.

#### Employment factors

Having to work versus not needing to work (e.g., being retired) was a cause of financial hardship. While many participants reported supportive work environments, it was clear this was not always the case. Provision of reasonable accommodations (flexible schedule, work from home, someone at work to compensate, flexible work tasks) was key to ensuring participants were able to work and maintain their income. Reasonable accommodations were protective regardless of whether they were provided for the cancer or another condition. This was often dependent on an understanding supervisor and/or coworkers who did not pressure the participants. Employment factors increased the level of financial hardship and financial worry for participants. Sometimes this was due to health insurance being tied to employment.

Participants experienced substantial administrative hurdles to access employment-related benefits such as Family and Medical Leave Act (FMLA), short term disability insurance, Americans with Disabilities Act (ADA) accommodations or Consolidated Omnibus Budget Reconciliation Act (COBRA) health insurance. For participants that did access these programs, the stress of the administrative hurdles was high. Some participants choose to forego the benefits simply because of the administrative hurdles or used paid time off (PTO) to avoid the application process and its attending burden and stress. One participant talked about the relief about not having to take on administrative burdens:

*“But you know, I think the- what you call like the red tape. If, you know, I had to do a short-term disability or you know leave of absence I'm sure I could have figured it out. But that's something I don't look forward to, even as a manager of people, you know occasionally have to, you know, dig into that and it's always frustrating so that's another frustration that I'm glad I avoided.”*

Being self-employed could be precarious due to lack of paid leave, or it could be protective if the participant had employees that continued to run the business. Long-term effects on employment included reduced earnings and delayed retirement.

#### Insurance factors

Insurance coverage was a major driver of financial hardship. Some participants had generous health insurance or multiple health insurance plans and experienced little financial hardship. Others had less generous or more difficult to access health insurance; these participants experienced administrative hurdles to cover care, such as needing to get care recoded by providers or appeal an insurance company decision.

Others struggled to balance working with receiving cancer care, particularly with maintaining employer-sponsored health insurance. Programs to maintain post-employment employer-sponsored health insurance were cost prohibitive or administratively burdensome. Other unhelpful aspects of health insurance were getting coverage for out of network care, multiple bills for the same episode of care, or providers that did not accept insurance that led to huge bills or inability to access care. Care that was often not covered or insufficiently covered included lymphedema, genetic testing, complementary and integrative medicine and reconstructive surgery. When talking about lymphedema, a common side effect of breast cancer treatment, one participant said:

*“And what I did find out is I tried to research was that if I needed, if I needed significant lymphatic massage, say through a massage therapist who's able to do that, nobody takes insurance for it. Nobody.”*

Problems with health insurance were not the only driver of financial hardship. Other forms of insurance such as life insurance and short-term disability insurance caused stress and financial hardship. Claims for short-term disability insurance were often denied, introducing an administrative burden to appeal. Overall, generous insurance buffered the best against financial hardship, while administrative hurdles created stress and potentially worsened financial burden.

#### Out of pocket care costs

Participants' out of pocket costs varied from small amounts to greater than $80,000 for one procedure. This included co-pays, deductibles and co-insurance. Sometimes participants were charged for care that would ultimately be covered by their health insurance or received multiple unexpected (surprise) bills; either scenario could create stress. Other out-of-pocket costs included items not typically considered medical care, such as sunscreen or special pillows; new clothes due to treatment-related weight fluctuations; or food costs during cancer care. The line between medical costs and living costs would sometimes blur. One participant reported:

*“But it's like they give you an itinerary and you're at [the cancer center] all day basically so you go down to the cafeteria because they have food that you can eat that's prepared for people that have had transplants very specific. You know specifications and preparation rules. And you just end up putting on a credit card. I mean that was one of our biggest expenses was not being able to go back to the apartment. To like make food? And ending up eating at [cancer center] because it's not inexpensive so. It's kind of an interesting rub that the place that's offering you the food you can eat when you need to eat it is so expensive.”*

Other costs included healthier food, care for side effects from cancer treatment, and office visits for each chemotherapy appointment. Health insurance characteristics such as deductibles and miscoding of care affected costs of care and whether participants sought care. Methods to reduce costs, such as finding out costs ahead of time to compare treatments or appeal denials of health insurance coverage, were often prohibitively time-consuming.

#### Living costs

Participants experienced financial hardship from living costs, including housing (e.g., rent, mortgage payments, yardwork, house cleaning), food, utilities and transportation, and pet care. Sometimes, the struggle to afford living costs could affect participants' health. One participant mentioned: *“I don't spend a lot, but I probably don't eat as well as I should because of the cost of food.”* While some participants did not struggle to afford living costs, others experienced extreme difficulty paying for necessities partially due to how much cancer and other medical care was covered by health insurance. Recreational activities such as family vacations were often no longer feasible due to increased out of pocket costs and/or reduced income.

### Dimensions of financial hardship

#### Financial coping

Coping actions ranged from minor rebudgeting to reducing spending on vacations to skipping or delaying healthcare (for cancer and/or other conditions) to delaying retirement and even major decisions about family structure, such as not having children, getting divorced or getting married. One participant stated: *“We consider[ed] getting divorced at one point to protect us financially.”* Other coping strategies included negotiating with insurance companies or healthcare providers to reduce costs; attempting to fit all care into a single calendar year; or taking less time off work then needed due to lack of PTO. The need to financially cope, and the time and burden required to complete coping actions, was often distressing even for those who successfully avoided financial hardship.

#### Financial consequences

Participants reported well-known financial consequences such as bills going to collections. Other financial consequences included needing to stay married due to finances, not being able to attend cultural practices due to costs (i.e., religious events, weddings), difficulty affording necessities and decreases in credit ratings. Some actions overlapped between financial coping and financial consequences. These included not saving desired amounts for retirement; draining savings or retirement accounts; delaying retirement; and staying with an unwanted job to maintain health or life insurance. Financial consequences were nearly always distressing for participants.

#### Financial depression & rumination

Financial hardship led to feeling stressed or upset separately from the other stressors of cancer. This included behavioral symptoms such as crying and physical symptoms such as stomach aches, shaking and tension. This was sometimes described as stress or feeling angry. When reviewing the draft items on financial depression and rumination, cognitive interview participants stated the items made sense and captured patients' experience.

#### Financial worry

Participants worried about paying for care and affording health insurance. The lack of transparency around care costs, coverage and timing of bills was often a source of financial worry: *“I was at my wits end. I was having bills come in and I didn't know what to do.”* Co-pays, out-of-network care, whether care would be covered and over-the-counter medications were also sources of worry. Financial worry also extended to living costs and maintaining income or employment during cancer care. Participants reported that having generous health insurance or income not dependent on ability to work reduced financial worry. Those who experienced financial worry also experienced impacts on sleep and physical symptoms.

### Context & individual factors

We examined how community, organizational and policy contexts influence financial hardship. We also examined how individual patient-level differences may influence the experience of financial hardship.

#### Positive support

Both formal and informal social support buffered against financial hardships. Formal support included American Cancer Society programs and emotional or informational support from healthcare providers. Even health insurance companies could provide support: *“The people from Medicaid were really wonderful, I mean they were super nice and helpful, so that made it a lot easier.”* Other formal support included social work and nutrition programs. Support from family and friends, providing tangible assistance such as money or groceries, buffered against financial hardship. Coworkers and bosses also provided support. These informal supports often filled the gap in financial needs left by lack of paid time off and insufficient health insurance coverage and could successfully prevent or mitigate financial hardship. However, in some cases, participants needed support but it was not provided.

#### Social & caregiving context

The support from or need to support partners could influence the degree of financial hardship as did the ages and needs of the participants' children. The nature of extended family relationships also impacted financial hardship. One participant reported: *“My family was not functional and so they were not available as resources for me.”* Some family members had their own illnesses and the patient ended up providing support and caregiving. Patients had caregiving needs themselves while balancing being caregivers to their own family.

#### Time & location

Cancer care introduces transportation and time costs that could be substantial. One participant said:

*“I went through [cancer care provider], and their clinics are- at that time-like now they have this big cancer clinic that my daughter went to. So I know the difference. But mine were all spread out at different places. The surgery I had, where I went to chemotherapy.”*

Participants observed that the factors that led to financial hardship from cancer had not improved over time, either when comparing their experience to others or comparing initial diagnoses to recurrences. However, changes in the participants' social and caregiving context could affect financial hardship over time.

#### Cancer symptom burden & financial hardship

The side effects of cancer treatment and symptoms of cancer appeared related to the level of financial hardship. For example, lymphedema, fatigue or cognitive symptoms prevented some participants from working at their pre-cancer levels. For others, the need to take off significant time from work negatively affected career trajectories, including job loss, voluntary and involuntary job changes, salary loss and lack of career advancement. Cancer treatment side effects and symptoms also increased the costs of care: *“The cancer treatment that likely related to having a later procedure did cause some financial hardship, not something super significant. But you know some.”* Treatment of lymphedema led to difficulty working and increased treatment costs. The stress from cancer and cancer treatments could limit participants' ability to cope with the administrative burdens of accessing disability and health insurance. Some financial effects continued well past active cancer treatment: *“we've had payment plans for medical bills for the last 13 years of our lives.”*

#### Luck

Multiple participants cited luck as a primary reason for not having worse financial hardship. Lucky circumstances were experienced as either random chance or as good fortune by receiving support, often through reduced administrative hurdles. Chance-related luck included not having worse side effects, COVID-19 providing an alternative reason for accommodations, being able to get all cancer care within a single calendar year or having good income before the cancer diagnosis. Favorable employment and insurance factors were also described as luck, such as working for an employer who decided to provide support and accommodation or having generous health insurance coverage that was easy to access. Support from family, friends, doctors and especially workplaces were cited as luck.

#### Individual factors

Participants' pre-cancer health and money habits appeared to be associated with their level of financial hardship. Pre-cancer health helped protect participants from worse financial hardship. Some people reported tending to save and others tended to prioritize experiences like family vacations, both before the cancer diagnosis and after the diagnosis. Other individual factors included awareness of paid sick leave or work leave policies.

### Effects of financial hardship

#### Non-financial consequences

Some participants were unable to adequately rest and recover from the cancer and cancer treatment. Financial coping behaviors, especially those that involved administrative hurdles, took large amounts of time, which further impacted rest and recovery. One participant said: “So I'm going to mention it, the amount of time I spent doing really basic things that should have been easy, on the phone advocating for myself”. Some participants were pressured to return to work sooner than they wanted by coworkers and did not get sufficient rest.

Other consequences included impacts on major life choices, such as forgoing having children. Some participants limited fun activities so paying bills and maintaining health insurance could be a priority. Others reported consequences of financial hardship included stress, ‘sheer hysteria’, worse health behaviors (e.g. less exercise due to energy going to work), physical symptoms and avoidance of finances.

#### Caregiver effects

Participants reported that caregivers, including spouses and adult children, also experienced financial hardship. Some had to take unpaid time off work while others worked more hours to increase income. Others had to quit school or jobs while other caregivers felt locked into a job: *“He was considering a job transition and he held off on doing that for a while.”* Some couples were buffered from worse financial hardship because both members of the couple were working and they had enough joint resources or the caregiver's employer provided PTO.

#### Health behaviors

Cancer diagnosis and treatment often motivated participants to exercise more and improve their diet. Other health behavior changes included taking precautions against COVID-19, sleep hygiene, being more involved in medical care, taking supplements and reducing alcohol use. However, a healthier diet often came with a greater cost that contributed to financial hardship and increased cost of living. The relationship between health behaviors and financial hardship was bidirectional.

### Revisions to the conceptual model & item banks

**Figure 1.**
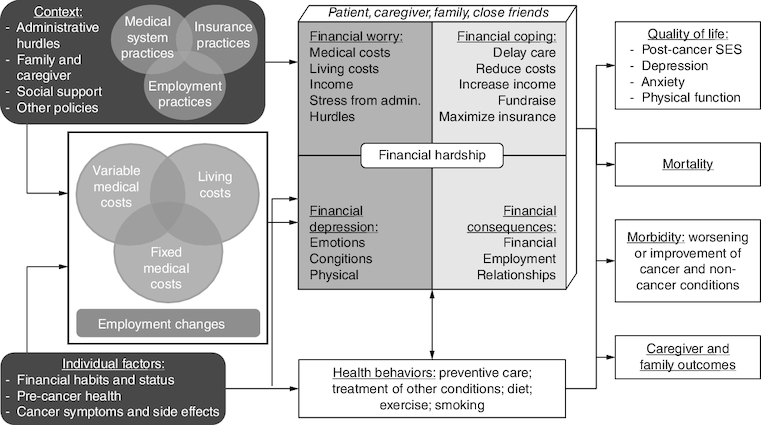
Revised conceptual model of financial hardship after cancer diagnosis. Data taken from [13].

The overall structure of the model (causal factors, financial hardship dimensions, outcomes affected by financial hardship) was maintained. However, based on the results of the interviews, we revised much of the content of the conceptual model and made a few structural changes. The most significant change focused on contextual and individual factors. In the previous model, these were not well defined and were grouped into two catchall categories of ‘context’ and ‘moderators’. The revised model (Figure 1 & Table 1) provides more specificity on several context factors influenced by organizational and government policy: medical system practices; insurance practices; employment practices; administrative hurdles; family and caregiver factors; and social support. Some factors (medical, insurance and employment practices) could interact such as medical coding affecting insurance coverage, insurance plan requirements influencing providers to be out-of-network or paid leave policies combined with locations of medical centers affecting access to care. Administrative hurdles are defined as additional paperwork or actions a patient or their provider must take to access benefits and spans across medical, insurance and employment factors. Social and caregiver context and social support highlight potential buffering or exacerbating interactions with context. As another change emphasizing the importance of caregivers (both the patient's caregivers and the patient's role as a caregiver), we added caregiver and family outcomes as another class of outcomes. These specific factors are highlighted because they were the most relevant in the interviews.

Individual factors were also revised to those most relevant such as financial status and treatment side effects (Table 1). However, it is important to note that even someone who has so-called ‘good’ individual factors such as financial habits (saves money, financially aware, monitors spending) can still experience financial hardship after cancer diagnosis.

We made minor changes to the causes of financial hardship (costs, employment changes) and to financial hardship itself. Categories of costs are now defined as overlapping due to some costs being both medical and nonmedical, such as needing new clothes or food. The four types of financial hardship are also conceptualized as overlapping and definitions have been expanded to include more coping behaviors, more types of worry, and more financial consequences. Morbidity, an outcome, has also been revised to clarify that this includes both cancer and noncancer aspects of health.

An addition to the model is health behaviors, actions people take that improve or worsen their physical or mental health (Figure 1 & Table 1). While diet and exercise were mentioned most frequently in the interviews, health behaviors can include smoking, alcohol use, preventive care and taking precautions against infectious diseases. The relationship between financial hardship and health behaviors is defined as bidirectional. Financial hardship can make it more difficult to engage in costly health behaviors and increasing positive health behaviors can worsen financial hardship due to increased living costs and negative health behaviors (smoking) can worsen financial hardship.

We revised the item banks assessing the four dimensions of financial hardship based on feedback from the interviews. We added several coping behaviors (checking health insurance claims, bartering) to the financial coping item bank. Items assessing the burdens associated with financial coping were added. Several specific financial consequences (quit college or school) that were reported in the interviews were added. Multiple items that participants worried about paying for were added to the financial worry item bank (scans/tests, over-the-counter treatments). The financial worry item bank was also expanded to include questions on worry about the effects of cancer on their employment. Based on the cognitive interviews, minor wording changes were also made. The number of items in each bank after revisions were: 42 items-financial coping; 22 items-financial consequences; 15 items-financial depression; 24 items-financial worry about healthcare.

## Discussion

This study used interviews of women with breast cancer and qualitative analysis to revise a conceptual model and begin to develop item banks to assess dimensions of financial hardship after cancer. Revisions to the conceptual model included the addition of administrative hurdles to accessing employment and insurance benefits as well as acknowledging the intersecting nature of medical and living costs and the overlap between dimensions of financial hardship. In addition to employment policies around sick leave and health insurance practices affecting financial hardship, the cause of financial hardship could be administrative hurdles and other people's reactions to the cancer. Luck was cited as a determinant of financial hardship and was oftentimes determined by a lack of administrative hurdles to accessing insurance or employment benefits. Another aspect of luck was cancer symptoms and side effects that healthcare providers could address. We created item banks for each of the four dimensions of financial hardship. The interviews led to several key new items among financial coping, financial consequences and financial worry. The addition of new items to the financial hardship measures that emerged from the interviews was surprising given the amount of previous research on this topic. Our work highlights the complex nature of financial hardship and how financial hardship can manifest in a variety of ways for each patient. Our next steps include evaluating the item banks in a larger sample of people with cancer using item response theory and testing some of the associations in the conceptual model (employment, insurance factors) in a longitudinal study. The revised conceptual model and item banks will be useful for future studies of financial hardship in cancer.

Our findings from the interviews were consistent with prior research which has shown the importance of employment and health insurance coverage in determining a patient's level of financial hardship [^29-32^]. However, our work extends prior research by identifying specific administrative hurdles to employment accommodations and insurance coverage such as excessive paperwork to access paid leave and denials of insurance coverage. It is important to note that while this study was conducted in the United States, financial hardship can still occur in countries with universal healthcare [33,34], partially due to uncovered care (out-of-networks/formulary) and lack of employment protections.

Our results suggest specific aspects of employment and insurance that could be changed to prevent or reduce financial hardship. Consistent with prior studies, our results support paid sick leave but also suggest reasonable work accommodations such as remote work and flexible work schedules and work tasks are key for reducing financial hardship. Our results also uniquely highlight that reducing administrative hurdles to using health insurance and short-term disability insurance could prevent financial hardship. It is important to note that administrative hurdles such as excessive paperwork, prior authorizations, out-of-network/formulary care and coverage denials made federal benefits programs meant to protect against financial hardship, such as FMLA and the ADA, effectively inaccessible to patients. Our qualitative results and the greater literature support a continued focus on employment and insurance to prevent financial hardship and highlight a need for a shift in focus toward reducing administrative hurdles to accessing benefits.

The study results also have implications for future research on financial hardship in cancer. Once the item banks have been quantitatively evaluated using item response theory, they can be used to provide generalizable results that can be easily translated into clinical practice. The revised conceptual model can guide policy research as well as patient-level interventions and implementation. Pending future studies evaluating the item banks, the measures and conceptual model will be useful tools for future studies on financial hardship after cancer diagnosis.

Our interview results suggest potential clinical changes to address financial hardship. Financial hardship screening may need to expand beyond financial consequences to include a focus on financial worry and financial coping and nurses and other healthcare personnel should be aware that financial hardship can manifest in a variety of ways. This could identify patients in need of early intervention and prevent severe financial consequences. Second, assessing both financial worry and financial coping could identify patients with more moderate or early levels of hardship who might benefit with additional support. Other potential patient-level interventions include care flow adjustments designed to minimize patient financial burdens [35]. For example, blood draws and appointments could be scheduled on the same day to save patients on parking and transportation costs; or patient parking costs could be waived. Once evaluated further, the item banks could be used to create brief screening measures of each dimension of financial hardship and to identify patients in need of social needs informed care.

In addition to financial hardship screening, several unique clinical implications were identified from the interview results. Financial hardship may have a bidirectional relationship with health behaviors such as exercise, diet and smoking. Clinicians should be aware of these relationships and help support patients in engaging in positive health behaviors in a way that does not exacerbate financial hardship. Given the important role of cancer symptoms and side effects in financial hardship, clinicians should also continue to encourage patients to share any potential symptom or side effect so healthcare providers can address these problems before they lead to financial hardship. The interview results suggest several avenues through which clinicians can use their skills to help prevent or reduce financial hardship.

The interview results imply several policy changes that could help prevent or mitigate financial hardship after cancer. Employment accommodations such as paid sick leave, remote work, and flexible schedules and work tasks could be required when feasible. Removal of administrative hurdles for health and disability insurance, such as requirements to streamline paperwork and processes for accessing benefits, could also help reduce financial hardship. Improving safety net programs are also another option and might involve small grants, unconditional cash transfers, or temporary aid that uses presumptive eligibility or does not require beneficiaries to be destitute before receiving help. As many participants experienced financial hardship due to the side effects of cancer treatment, patient-focused drug development and valuation should account for the additional financial burden associated with the side effects of treatment on patients and their caregivers [36]. Little is known about the contribution of out-of-network/formulary care to cancer patient's out of pocket costs and financial burden. Additional research is needed to quantify their impact and develop policies to reduce their burden on patients. Overall, a combination of policy changes and patient-level interventions could help reduce or even prevent financial hardship after cancer diagnosis.

The findings should be considered within the context of the limitations. This was a small interview study of only female breast cancer patients. Results might differ for men or people with other types of cancer. All participants were predominantly White and highly educated from USA in a state with a very high cost of living. Our results cannot speak substantively to the unique experiences of people with cancer from other racial and ethnic groups. It will be important in future research with a more diverse sample to explore different issue that may affect these groups. Given the unique insurance and employment ecosystem of USA, results might not apply to other countries and specific US populations. We used a framework analysis that was based on the previous model; despite the strengths of this approach in rapid thematic analysis and our identification of emergent themes such as luck and administrative burden, it is possible other emergent themes could have been missed. The strengths of the study balance out these limitations and include a comprehensive assessment of financial hardship, rigorous analyses and development of a conceptual model with corresponding measures.

## Conclusion

Our results highlight several contextual factors relevant to progress in addressing cancer-related financial hardship. Our results support patient-level interventions with a broad focus that includes strategies to use disability insurance and successfully obtain employment accommodations in addition to current approaches focused on health insurance and referral to financial aid. Findings suggest several unique directions for future research and for policy and clinical interventions, including policy changes reducing administrative hurdles to reasonable employment accommodations and insurance benefits. Screening measures for financial hardship in cancer care may require screening for multiple dimensions of financial hardship and its consequences. Interventions for screen-detected financial hardship should likely include guidance on negotiating the administrative burden of benefits. Financial hardship remains a substantial problem after cancer diagnosis and future research, clinical and policy work is needed to address this public health issue.
